# Protecting Future Generations From Hereditary Genetic Disorders Using Preimplantation Genetic Diagnosis: A Narrative Review Article

**DOI:** 10.7759/cureus.75465

**Published:** 2024-12-10

**Authors:** Farah M Zolaly, Amal Y Zaman, Shaden M Hazmi

**Affiliations:** 1 Medicine, College of Medicine, Taibah University, Medina, SAU; 2 Obstetrics and Gynaecology, Taibah University, Medina, SAU

**Keywords:** contiguity and genetic disorders, hereditary disorders, new techniques for preimplantation genetic diagnosis, preimplantation genetic diagnosis, preimplantation genetic diagnosis indications, saudi arabia

## Abstract

Preimplantation genetic diagnosis (PGD) is provided by majority of reproductive clinics in the United States (US), and PGD is used in many in vitro fertilization (IVF) procedures every year. PGD is extensively used to screen for certain genetic abnormalities and aneuploidy in individuals undergoing IVF. Genetic disorders are very prevalent in Saudi Arabia. The high prevalence of consanguinity in the population explains, to a great extent, the incidence of genetic disorders being relatively higher in the Kingdom of Saudi Arabia (KSA) compared to many other countries. Estimates range from 6% to 10% among all live births, and at least one study estimated that up to 30% of the population couldbe affected by a genetic disorder at any time in their lifetime. Therefore, a preventive measure such as PGD is useful for detecting genetic disorders before implantation. In KSA, genetic disorders are prevalent with a high psychological and financial burden.

A diagnostic procedure like PGD is useful for detecting genetic disorders. It is used to analyze embryos under the IVF cycle. Then, a biopsy is taken from the embryos and analyzed using techniques such as polymerase chain reaction (PCR) and fluorescence in situ hybridization (FISH). Only healthy embryos are transferred to the uterus, thus eliminating the need for a parental diagnostic test that comes with substantial risk to the mother and the fetus. PGD is used for the detection of genetic abnormalities such as sex-linked diseases and chromosomal abnormalities, helping parents with genetic disorders to conceive a healthy child. Some of the limitations of PGD are its unavailability and the high cost, which limit the access for parents of low socioeconomic status. It creates many decision-making problems for the facilities and parents.

Our objectives include giving an overview of PGD history, demonstrating indications for this procedure, explaining its techniques, and discussing the ethical issues surrounding it. PGD offers improved IVF results and reduced genetic disorder prevalence but also has disadvantages like high costs, limited access, and ethical concerns.

## Introduction and background

Preimplantation genetic diagnosis (PGD) is an established procedure for embryo genetic analysis via a cycle of in vitro fertilization (IVF), where a single cell that was biopsied from the IVF embryos before implantation is evaluated and examined for genetic diseases or chromosomal abnormalities in the genetic composition. This is very important, mainly because of the high burden of genetic disorders on the health system, especially in countries where consanguinity marriages are common, like in Arab countries [1,2].

PGD is a recognized and valid alternative to classical prenatal diagnosis in families with inherited disorders worldwide. It is a known fact that families with congenital diseases are prone to having affected children who can suffer from short- and long-term health consequences, resulting in up to 50% of offspring being affected [1]. It can help couples who are affected or are carriers of genetic diseases, which puts them at a higher risk of conceiving an affected embryo, which can only be diagnosed via amniocentesis, a procedure that has many risks and may result in a miscarriage. PGD allows a normal pregnancy by ensuring the transfer of only unaffected embryos [2].

The main indications of PGD are single-gene disorders, chromosomal aberration like translocation, recurrent miscarriages related to chromosomal aberration, and recurrent implantation failure. PGD has an advantage in avoiding pregnancy termination and related complications in mothers with a high-risk pregnancy. Thus, by performing this procedure, we can spare the parents the psychological and social impact of a miscarriage and pregnancy termination. The prospective study by Geraedts et al. recruited 186 women. 128 participants had a diagnosis of early pregnancy loss (EPL), and 58 had ongoing pregnancies. The result showed that there was an increase in psychological morbidity in the EPL group [2]. During the first month following the pregnancy loss, 28% of the participants in that group met the criteria for probable post-traumatic stress disorder (PTSD), 32% for anxiety, and 16% for depression. Furthermore, at three months, 38% meet the criteria for PTSD, 20% for anxiety, and 5% for depression [3]. While in the control group, no women met the criteria for PTSD, and only 10% met the criteria for anxiety and depression. This clearly shows that a significant percentage of women suffer from psychological stress that can be prevented by PGD [2]. Another used application for PGD is aneuploidy. Screening in assisted conception will improve infertile patients' chances in the advanced maternal age, even though the level of its efficacy is yet to be established. In addition, the use of PGD has extended to other indications, such as genetically predisposed late-onset diseases and human leukocyte antigen (HLA) typing for stem cell transplantation [2].

After IVF, the material is obtained from an oocyte (polar body), a three-day embryo, or, more commonly, from a blastocyst trophectoderm. The choice of the diagnostic method depends on the testing center. Still, the methods with the highest reliability and precision are comparative genomic hybridization array (aCGH) and next-generation sequencing (NGS), since both enable comprehensive genetic analysis of an embryo. aCGH is very effective in screening for larger chromosomal abnormalities, and NGS provides resolution down to the base pair level, a facility that is required for detailed genetic diagnoses, which also identify specific mutations that cause hereditary diseases. All these technologies taken together contribute to the success of PGD by increasing its accuracy of genetic assessment, reducing the risk of transferring embryos with genetic disorders and therefore offering better chances for a successful pregnancy and healthy offspring [4].

The major limitation of some types of genetic testing used for PGD include the fact that not all different types of genetic mutations may be approached with a single method. Different genes have unique variations, and thus, each may require a specific form of testing to identify any issues. This is especially a concern in the PGD, as only minimal DNA can be taken off embryos, and results are often needed in a relatively short period if parents wish to transfer an embryo immediately. Therefore, developing tailored tests for each mutation, while needing fast results, can be complex [5]. PGD has been involved with substantial controversy as a reproductive technology concerning embryo selection. The fetus's status and the desires and responsibilities of the parents are important ethical concerns. In terms of social policy, taking account of the access issues and the effect that the technology has on families, women, and the physician's duties. The impact of using sex selection and selecting an embryo unaffected by a serious disorder should be considered [6].

## Review

Consanguineous marriages and genetic disorders in Saudi Arabia

The Prevalence of Consanguinity and Genetic Disorders in Saudi Arabia: Regional Variances and Impact on Offspring

Consanguineous marriages, especially those with known genetic diseases, are at a higher risk of acquiring offspring with genetic damage when compared to the general population. Thus, the rate at which genetic diseases may occur is higher among consanguineous marriages. According to the total consanguinity rate, 57.7% of the screened families were consanguineous. First cousin marriages accounted for 28.4% of all marriages, with distant relative marriages coming in second with 15.2% and second cousin marriages with 14.6%. Samtah had the highest consanguinity rate at 80.6%, According to these findings, Saudi Arabia is one of the nations with the highest rate of consanguinity in the globe. This is important as high prevalence of consanguinity and many genetic disorders coexist [7].

There was documentation that in KSA the prevalence of consanguinity differs within various regions; it was highest on the north-western side of KSA, particularly in Khaibar, Makkah, and Taif, estimated at up to 67.7% [5]. In the province of Almadinah Almunawwarah, approximately 60-67% of marriages were found to be in consanguine couples. This percentile is highly greater, reaching >80% in rural KSA areas while Abha in the province of South Western had the lowest percentage at about 34% [8].

Addressing Consanguinity in Saudi Arabia: The Role of Women's Education and Cultural Factors

Women's education reportedly resulted in a decline in consanguinity; however, consanguineous marriages may be encouraged by a number of underlying factors, such as cultural standards, economic considerations, societal structures, traditional traditions, and the desire to maintain property within family, which can be ascribed to Saudi Arabia's high percentage of consanguinity. These rates, therefore, would be further reduced only by specific suitable interventions. These may include community-level educational programs, where families are counseled on the risks that come with marrying a relative and also the advantages of marrying outside the family.

The study by El Mouzan et al. considered more details of some genetic disorders in the KSA, such as the prevalence of Down syndrome, which was reported to be 1.8 per 1,000 live births [7]. Other studies concerning hereditary hematological diseases in Saudi Arabia usually take place in different regions of Saudi Arabia. One of them is sickle cell disease (SCD), endemic in some areas of the country, with a prevalence ranging between 91 and 99 per 10,000 live births. There are also areas with an increased prevalence of glucose-6-phosphate dehydrogenase (G6PD) deficiency, with a value of 20 per 1,000 births. The prevalence of sickle cell anemia and beta-thalassemia in Saudi Arabia was 49.6 and 13.6 cases per 1,000 people, respectively. Along with beta-thalassemia and SCD, alpha-thalassemia is also very common, with rates ranging from 5.9% in the eastern region to 0.4% in the northern region [7]. However, the relation between different genetic diseases and consanguinity was studied and proved to be statistically significant for major congenital malformations and congenital heart diseases (CHD) [7].

History of PGD

Evolution of PGD: Historical Milestones and Early Applications

It was only in 1968 that PGD was initiated when Edwards and Gardner took two cell biopsies and done Barr body sexing on a rabbit embryo blastocyst. Then in 1978, Edwards and Steptoe did the first and successful IVF and thus made PGD as a possible in humans. The first clinical uses of PGD were documented in 1990, when the first pregnancy was achieved in couples at risk of having children with X-linked disorders. This was done by embryo biopsy and sex determination by PCR. Since then, an increasing number of PGD cycles has been utilized [9]. PGD is a procedure that has been established for the genetic analysis of the embryo via a cycle of IVF.

PGD was developed almost 25 years ago as an alternative to prenatal diagnosis carried out on embryos. This was initially provided for diagnosis in parents susceptible to single-gene disorders such as cystic fibrosis, spinal muscle atrophy, and Huntington's disease. PGD has also become more used in the area of assisted reproduction for chromosome aneuploidy detection owing to advancing maternal age or chromosomal abnormalities [10].

Advancements in PGD Techniques: From Single-Gene Disorders to Chromosome Abnormalities

The early study of PGD goes back to Edwards and Gardner, who biopsied a rabbit blastocyst in 1968 and performed a Barr body analysis to determine the sex. The first active IVF by Edwards and Steptoe in 1978 made PGD possible in humans. More studies in PGD into blastomeres have been carried out within the last decade, and in 1988, Monk and Handy were able to apply PGD to identify a single genetic disorder. The first successful application of PGD on humans was done by Handyside and colleagues in 1990. They have used PCR to perform sexing embryos to detect the existence of the Y-chromosome sequence in order to avoid males that are affected with X-linked adrenoleukodystrophy and X-linked mental retardation. Later on, effective PGD for cystic fibrosis, α-1 antitrypsin deficiency, and a number of other gene-related diseases were reported. The further extension of the use of fluorescence in situ hybridization (FISH) in biopsied blastomeres by Griffin and Griffo expanded the range of PGD uses to include chromosome abnormalities in embryos, improving tied structural rearrangements such as translocations and inversions, their alterations/deletions, and also age-related aneuploidy in order to improve the outcome of IVF. After Griffin and Giriffo, who for the first time used chromosome X and Y probes, the development of PGD technology did not stop. Later, Munne developed multicolor FISH protocols allowing the screening of up to five chromosomes simultaneously. Then, FISH was largely replaced by molecular aneuploid testing approaches, including aCGH, single nucleotide polymorphism (SNP) arrays, quantitative polymerase chain reaction (qPCR) and NGS. Future uses of PGD have grown significantly with the remarkable capacity of modern sequencer systems to produce vast quantities rapidly burden of genetic disorders on health system, especially in countries where consanguinity marriages are common like in Arab countries [10].

Selected procedures of PGD

Preparation of the Procedure

Before PGD is initiated, genetic counseling must be provided by a licensed genetic counselor to ensure that patients ultimately realize the risk of raising an affected child, the effect of the condition on the child, and the advantages and disadvantages of all current methods for preimplantation and prenatal evaluation. The procedure of PGD requires close cooperation between obstetricians, infertility specialists, and geneticists. Geneticists interpret genetic material for abnormalities, the infertility specialist conducts the process in the line of IVF and embryo selection, and the obstetrician does the follow-up care in the pregnancy. Couples should be told that PGD will minimize the probability of conceiving a child with a genetic abnormality carried by one or both parents if that abnormality can be detected with tests conducted on a single cell or on several trophectoderm cells [11]. It requires concerted commitment, costly techniques, and exhaustion for the patients, but it provides enormous potential for couples whose previous child has displayed genetic abnormalities [12].

Techniques of Biopsy

Polar body biopsy: A polar body biopsy could be performed by collecting the first and/or second polar bodies that are extruded from the oocyte and investigating them through genetic examination for studying the particular maternal involvement of the embryo and to detect chromosomal translocations or genetic mutations. Polar body biopsies may be performed as a pre-implant diagnostic tool for the diagnosis or prevention of fertilization and transition of the oocyte with common aneuploidy in advanced-age IVF patients [1].

The advantages of polar body biopsy provide information on maternal chromosomes and thus allow for the identification of chromosomal abnormalities, translocations, and genetic mutations that could affect the viability of the embryos. Also, this may also serve as a means of preimplantation diagnosis, especially in cases of older-age IVF patients who bear greater risks for aneuploidy, in order to choose healthier embryos.

Furthermore, because polar bodies are extruded and discarded, the procedure may involve less manipulation of the embryo, thus preserving its integrity. For the limitations polar bodies provide information related only to the maternal genome and no information about the paternal genome that may lead to incomplete genetic profiling of the embryo. Also, polar body cannot be representative of the whole embryo genetic material; thus, there may be a chance of false positives or false negatives as to genetic testing. Furthermore, the timing for performing the biopsy is very critical, being performed at very specific stages of oocyte maturation that could contradict clinical protocols.

Trophectoderm biopsy: Contrarily, trophectoderm biopsy does not significantly affect embryo development when compared to blastomere biopsy. Therefore, trophectoderm biopsy should be the preferred method, where applicable, in embryo biopsy and should be done by trained hands. For all PGD cases, it is recommended that intracytoplasmic sperm injection (ICSI) be performed to eliminate the risk of sperm attachment to the zona pellucida causing paternal contamination.

Embryo biopsy by a double needle approach was performed at 6-10 cell-stage embryos, three days at the eight-cell stage post-insemination, in Ca2+/Mg2+ free medium under oil self-administered gerocognitive exam (SAGE). This technique is done by using one of the three methods mentioned to open the zone pellucida; it is done either by a chemical, laser, or mechanical method. To the uterus, only healthy embryos are moved. This is due to the fact that the ovarian reserve needs to be optimal since an adequate number of embryos is, above all, essential to reach.

The advantages of this are trophectoderm biopsy obtains maternal and paternal contributions, hence, the evaluation of the entire genetic status of the embryo can be performed.

For the limitations though the effect on embryo development is minimal, any form of trophectodermal biopsy is still considered invasive and may bring additional risks or complications. Technical expertise required, the end if the biopsy rests in the hands of skilled embryologists who are busy looking after the embryos so that the biopsy is not performed in a way that damages the embryo. This is still an invasive procedure and because of how complicated the procedure is, its availability may be limited in some settings.

Role of ICSI in PGD

ICSI is employed for the intensification of success rates at the time of PGD by reducing paternal contamination while the embryo is undergoing a genetic test. It chooses healthy sperms and brings about controlled fertilization that the status of genetic analysis is true for the embryo. The synergy of ICSI with PGD enhances the precision of genetic testing and empowers the couple to make more informed decisions regarding embryo transfer, thus improving the chances of successful pregnancies and the birth of healthy offspring.

Paternal genetic material reduction: ICSI, in many ways, minimizes the chances of paternal contamination of genetic analyses. In a normal setting of IVF, it is assumed that there is a potential for the sperm to carry on genetic abnormalities, and the presence of sperm-derived material in the embryo could complicate genetic assessments. Using ICSI allows only the selected sperm to be introduced into the oocyte; fertilization, therefore, is much more controlled, and the risk of any unwanted paternal genetic contributions confounding the results is low.

Improved genetic results: ICSI makes it possible to choose the healthiest sperm, which can sometimes increase the chances of producing genetically typical embryos in some cases. This is mainly due to PGD as the transfer of healthy embryos tends to reduce genetic disorders in offspring further.

Specific genetic testing: When specific genetic disorders are being tested, ICSI gives the ability to include exactly the genetic material arising from the oocyte. This is especially so in couples where PGD is done because the disorders may include a paternal element; this would mean that the genetic diagnosis truly reflects embryo viability and genetic integrity undisturbed by sperm-related problems.

Embryo Transfer and Amniocentesis

Once embryos have been chosen based on compatible signals via FISH, chosen embryos are transferred into the patient's uterus. This step can only happen on the fourth or fifth day after the retrieval of oocytes. This is a critical stage for ensuring that embryos devoid of any detected genetic abnormality are implanted to increase the chances of successful pregnancy. The next examination will be amniocentesis in clinically pregnant women to confirm the results of PGD and complete the genetic assessment of the embryo.

Multiple complications could arise at this stage, such as failure of implantation since the rate of successful implantation in PGD is lower than the rate of classical IVF; in contrast, PGD could provide a solution and explanation to patients with recurrent IVF failure [13].

Chromosomal Analysis

For this reason, PGD is important in the diagnosis of chromosomal anomalies in parents at an increased risk of chromosomal abnormalities due to advanced maternal age or unbalanced paternal karyotype chromosome rearrangements before implantation. Being an advanced diagnostic modality, it finds genetic problems in embryos conceived via IVF. It provides a way to prevent certain disorders that otherwise may have been diagnosed after birth or later in life through traditional prenatal diagnosis, such as trisomies and aneuploidies, and for couples with a balanced chromosomal translocation or carriers of recessive diseases such as cystic fibrosis or SCD. All these detectable abnormalities cause incurable diseases with a need for lifelong treatments, possible disabilities, and poor quality of life.

PGD decreases the necessity of prenatal diagnosis and the subsequent option of aborting a pregnancy based on the results of genetic disorder detection in an embryo. Furthermore, PGD may raise implantation and conception rates for childless couples by selecting embryos free from detectable genetic abnormalities, thus leaving more chances for the establishment of a healthy pregnancy.

This approach ensures better reproductive outcomes while giving each family control and peace of mind about their genetic health and future offspring. Although PGD is a useful tool for couples with genetic disease or recurrent miscarriages, PGD could be used for sex selection and inappropriate control over nonessential characteristics of children, which results in unnecessary medical burdens and costs for parents.

PGD techniques

PGD is done by IVF, followed by various genetic tests like NGS, qPCR, FISH, and comparative genomic hybridization (CGH), along with biopsy of the embryo at various stages of embryological development. Testing is performed on the availability of the facility. However, techniques like aCGH and NGS have the highest reliability [4]. Preimplantation genetic screening, or PGS, can be conducted in different cell types during preimplantation development: polar bodies from oocytes and zygotes, blastomeres from cleavage-stage embryos, or trophectoderm cells taken from the blastocyst [4]. In performing PGD, during IVF procedures, embryos are produced by fertilizing an egg with a sperm in the lab [4]. After that, different biopsy methods are used in different cell types, such as polar bodies used in early-stage biopsies focusing on them for genetic analysis. Blastomeres are collected on Day 3 (D3). This involves removing one or two cells from a preimplantation embryo, usually resulting in better outcomes when fresh transfer occurs the same day or on D4/D5. Another method is trophectoderm biopsy on D5/D6, where a biopsy of cells is taken from the trophectoderm of blastocysts. Finally, blastocentesis is a newer, less invasive technique aiming to extract cells and DNA from the blastocoelic cavity, showing promise for reducing the risk of embryo loss. All recognized biopsy methods are invasive and carry a risk of embryo loss. While they do not guarantee the embryo's proper constitution, they help minimize the risk of specific disorders.

The biopsy techniques also differ. Before biopsy, the zona pellucida must be perforated using mechanical methods such as cutting with a micropipette, chemical methods using an acid solution to dissolve the zona pellucida, or laser methods using a laser beam modulated through a microscope. The biopsy procedure goes through stages starting with preparation by placing the embryos in a medium to loosen cell junctions and separate them in micro-drops under oil. Then micromanipulation takes place by focusing on the embryo at 400X magnification and securing it with a micropipette holder. Finally, the biopsy execution is done by perforating the zona pellucida and gently removing the required cells, such as polar bodies, blastomeres, etc. It is important to note that for D5 biopsies, the zona pellucida is pierced on D3 to facilitate the blastocyst's hatching, making it easier to extract trophectoderm cells. After the biopsy is taken, the collected cells are processed according to the genetic study type. For example, cells are fixed on a slide for FISH studies. On the other hand, cells are placed in a small tube for PCR essays [14].

PCR

Challenges and solutions in PCR-based PGD: PCR was among the few early techniques employed for PGD. It helps in the determination of the sex of an embryo through amplifying a sequence of DNA on the Y chromosome. Thus, it helps in the avoidance of transferring X-linked disorders. DNA is released from a lysed cell, and the mutation site is magnified to a detectable level and analyzed for the presence or absence of mutation in the amplified DNA. Its greatest risk to diagnostic accuracy is contamination with extraneous DNA [15]. However, maternal contamination could be avoided by ensuring that all cumulus cells are completely removed from the oocyte before biopsy by gentle mechanical methods or enzymatic treatments. To identify potential contamination, negative controls in each batch of tests need to be included. Or by monitoring for contamination by using polymorphic microsatellite markers alongside disease-specific assays to detect unexpected alleles that could indicate contamination. Another risk to PCR diagnostic accuracy is the allele dropout (ADO), a single-cell PCR-specific event due to a failure to amplify one of the two alleles in a heterozygous cell that could lead to misdiagnosis and the transfer of an affected embryo [15]. Also, PCR primers may preferentially bind to one allele due to slight differences in sequence or secondary structures, leading to the amplification of only one allele. Another factor is the lysis process; it may not yield intact DNA, and any degradation can affect the amplification, making it more likely that one allele is missed. Technical variability plays a role because single-cell procedures are sensitive to variations in reaction conditions (e.g., temperature, time), which can impact the amplification efficiency and lead to inconsistent results. Haploidy In some cases, the biopsyed cell may be haploid for a specific locus, inherently causing ADO. These factors combined make single-cell PCR more prone to ADO compared to PCR performed on larger DNA samples, where the likelihood of amplifying both alleles is higher [16].

Current applications of PCR in PGD: PCR remains a critical technique, especially for specific applications. However, newer technologies have enhanced diagnostic capabilities and reduced certain risks associated with PCR. Offering more reliability and broader applications in genetic diagnosis. Conversely, PCR is still widely used in PGD, particularly for diagnosing monogenic diseases. PCR remains a primary method for analyzing genes associated with single-gene disorders. Its ability to amplify DNA from single cells makes it suitable for early diagnosis in embryos. It is also used as a complementary technique for newer methods, such as FISH and CGH, which are frequently used alongside PCR. These methods provide additional information, such as chromosomal integrity and aneuploidy screening, which PCR alone cannot offer [17].

FISH

It was used for the first time in 1992 as a means of sex selection for the prevention of X-linked recessive diseases, and then it was used all over the world in the testing of structural and numerical chromosomal abnormalities in metaphase chromosomes and interphase nuclei [18]. Numerically, some of the anatomical defects of the chromosomes in the embryo could be detected with the help of FISH. The probes usually utilized are those for chromosomes 13, 18, 21, X, and Y. Further, in instances of one parent being a carrier of translocation anomalies, FISH is also utilized for PGD [19]. This diagnostic tool combines conventional cytogenetics with the technology of molecular genetics.

Limitations and considerations of FISH in PGD: FISH faces many limitations, such as chromosomal mosaicism. This can lead to inaccurate assessments and false negatives results. Also, FISH typically analyzes only a limited number of chromosomes (usually 7-12). This selective analysis may overlook other significant chromosomal abnormalities that could impact embryo viability. FISH often focuses on the most common aneuploidies, which means rarer but clinically relevant abnormalities may not be detected. Another important limitation is the biopsy risks and their impact on embryo viability. The process of biopsy necessary to obtain cells for FISH can compromise the embryo's integrity and viability. The procedure itself may cause mechanical damage or stress to the embryo. Therefore, embryos subjected to biopsy may have lower survival rates post-transfer compared to non-biopsied embryos. Also, FISH is limited in its ability to provide a comprehensive assessment of the embryo's chromosomal health. This limitation can lead to an overestimation of the viability of embryos, especially in cases with high aneuploidy rates. Due to these factors, the potential for misdiagnosis is notable. Therefore, fish has been replaced as the gold standard by newer, more reliable technologies [20].

Expanding applications and indications for FISH in genetic testing and counseling: Among the signs where FISH may be helpful are sperm aneuploidy screening that is used in infertile men, which has prognostic significance. However, sperm aneuploidy testing has been of limited benefit because of technical difficulties and/or the cost. High cost of multiple FISH probes and technical difficulties of the test lead to avoidance of the use of sperm aneuploidy testing [21]. Also, FISH is particularly valuable in cases of complex translocations where multiple segments may be involved. It can help clarify the genetic status of embryos and provide more comprehensive information about structural rearrangements. FISH is often used as a follow-up to G-banding to provide a more detailed description and confirmation of the translocation. This is essential for ensuring the accuracy of genetic testing and counseling [22].

CGH

On the other hand, it has been successfully applied to polar body cleavage-stage embryos and blastocysts, with published precision ranging from 98% following blastomere biopsy and up to 95% following blastocyst biopsy [18]. This test is performed to identify the duplication or deletion of fragments of chromosomes without culturing the cells. It depends on the usage of tested DNA. CGH can detect minor differences in copy number variations that might be missed by other methods, such as SNP arrays. CGH arrays typically provide better signal-to-noise ratios compared to SNP arrays, enhancing the reliability of detected variations. High-density CGH arrays can be rapidly synthesized and customized to target specific genomic regions of interest, including complex and repeat-rich areas. CGH can analyze the entire genome, providing a broad overview of genomic alterations across multiple samples. Also, it is effective in identifying various structural variants beyond just SNPs, including larger insertions, deletions, and duplications [23]. The major disadvantage of typical CGH is its low resolution; this strongly affects its ability to detect smaller chromosomal abnormalities.

NGS Techniques

It is one of the massively parallel sequencing (MPS) techniques that allows the parallel processing of millions of nucleic acid molecules. This NGS technique has appeared as one of the superior alternatives of aCGH in PGD [4]. NGS can be used for all kinds of genetic scanning by both fresh and frozen cycles. It is the only process that allows for the analysis of chromosome aneuploidy or translocation and mutations responsible for any single-gene disease by using one biopsy and one method. NGS ability to detect single-gene disease combines one of the few remaining advantages of PCR with the advanced capabilities of newer technologies like aCGH. So, with the use of NGS, it becomes possible to investigate one entire genome with different abnormalities. This technology thus helped to reduce the cost of a single analysis, as the analysis of many samples at the same time became the most favorable method currently [4]. This is done by utilizing DNA barcode technology, NGS is capable of analyzing multiple samples from various patients on a single sequencing chip. These samples can represent different conditions, such as recurrent miscarriages, repeated implantation failures, and past aneuploidy cases.

Challenges and advancements in NGS technology for PGD: There is several limitations of NGS in preimplantation genetic testing need to be addressed. Firstly, while NGS-based comprehensive chromosomal screening offers considerable advantages for many IVF-PGS patients, it may not be suitable for all, particularly those with reduced ovarian reserve. Secondly, NGS is unable to directly identify balanced chromosomal rearrangements because there is no overall DNA content imbalance. Lastly, whole genome amplification (WGA) products have not been clearly defined for unbalanced translocation breakpoints, indicating a need for further research using cell lines or WGA products to improve detection of imbalanced translocations. Overall, NGS-based PGS has the potential for effective clinical application [24].

Comparative analysis of NGS and CGH in preimplantation genetic testing and counseling: NGS is capable of detecting unbalanced chromosome segments smaller than the manufacturer’s quoted resolution of ≥20 MB. Achieved a detection rate of 97% for unbalanced segments previously identified by array-CGH. Also, NGS showed 100% clinical outcome concordance for the embryos tested, meaning that despite some undetected segments, the overall diagnostic conclusions were consistent with clinical implications. A retrospective study of WGA products from 200 embryos known to carry unbalanced rearrangements indicates that array-CGH was able to detect certain rearrangements that NGS missed, particularly those <10 MB.

Some segments, particularly those <10 MB, were not detectable or presented issues with detection reliability. array-CGH remains more sensitive for some smaller rearrangements, particularly in acrocentric chromosomes, which NGS struggled to analyze due to lack of data points [25]. NGS can accurately detect mosaicism and segmental mutations, offering a high resolution for identifying chromosomal abnormalities. Its sensitivity in detecting mosaicism provides an advantage over CGH. NGS results can typically be produced in three to seven hours for up to 24 samples, making it relatively fast. NGS is more rapid than CGH, but more expensive and less widely available than it. NGS has shown improved ongoing pregnancy rates (OPR) and live birth rates (LBR) when compared to CGH, as it can more efficiently identify embryos with reduced viability. Finally, both NGS and CGH have their respective strengths and weaknesses [26].

Indications of PGD

According to a retrospective study by Abotalib conducted in Saudi Arabia, with a sample size of 137 patients and a total of 802 biopsied embryos, the most frequent use for PGD was sex-selection (57.7%), followed by repeated fetal loss (24.3%) and persistent IVF failure (13%) [1]. In several IVF cycles, PGD was done to detect embryos with structural and numerical aberrations of the chromosome [1]. In this study, 36.2% of the embryos had chromosomal abnormalities, 12.2% of the embryos were abnormal sex chromosomes, 3.2% were trisomies, and only one tetraploid was found [1]. The study results mentioned above are consistent with another retrospective cohort study by Plachot consisting of 34 cases from six different countries under the auspices of the European society of Human Reproduction and Embryology [16]. It found 4.8% trisomies in abortions in spontaneous pregnancies; the percentage increased in IVF pregnancies to 6% [16]. Chromosomal abnormalities such as aneuploidies were thought to be responsible for implantation failure, miscarriage, and birth defects [16]. Another common indication is for recurrent pregnancy loss. PGD is used to reduce the risk of a miscarriage and to avoid the trauma, pain, and side effects of recurrent miscarriages, even though there is a good chance of getting pregnant after standard treatment [17]. Another strong indicator for PGD is infertile patients. It has decreased trisomy pregnancies substantially regardless of maternal age and the number of produced embryos. It has also been shown to decrease significantly unintended abortions in infertile couples undergoing IVF [17].

Infertility, Single-Gene Disorders, Chromosomal Anomalies

Utilizing PGD for aneuploidy assessment to enhance IVF results in couples with recurrent pregnancy loss or unsuccessful IVF was the most common indication. Many clinics indicate that they use PGD for couples at increased threat of autosomal single-gene disorders, such as Tay Sachs disease, SCD, and myotonic dystrophy [10]. PGD provides multiple advantages over invasive prenatal diagnosis (chorionic villus sampling or amniocentesis), such as the These invasive tests are associated with a 1% risk of miscarriage. Associated with invasive prenatal diagnosis, other options, such as non-invasive prenatal diagnosis, have a high rate of false positives at 8%. Thus, alternative options available to avoid giving birth to an affected child include a change of partner, use of donor gametes, adoption, or opting to forgo having children altogether [27]. PGD in single-gene disorders has improved significantly over the last two decades, as it evolved to detect common Mendelian diseases, including myotonic dystrophy, Huntington's disease, FRGIL X syndrome, spinal muscle atrophy, cystic fibrosis, beta-thalassemia, and SCD [18]. Other clinics also offered PGD to prevent the beginning of adult diseases, such as Huntington disease, hereditary breast cancer, and Alzheimer's disease. Getting a genetic mutation correlated with a specific disease, such as hereditary breast cancer, does not mean that the child will develop the condition in the future, so children with adult-onset disorders may expect to stay healthy for a long time before symptoms begin [10].

Polygenic Disorders

More recently, PGD has also shown promise with a polygenic disorder sometimes known as "non-Mendelian," where phenotypes or characters depend on several loci. Variance outside of the nuclear genetic loci for most non-Mendelian diseases plays an etiological or phenotype-influent function such as epigenetic modifications, mitochondrial (and mDNA) modifications, post-translation modifications, and environmental contributions.

One of the only cases in which PGD is useful in non-Mendelian or polygenic diseases is where the incidence in different genders is uneven. For example, chances of diseases such as breast cancer, rheumatoid arthritis, and multiple sclerosis increase significantly, and a choice for a male embryo reduces the chance of devolving such a disease later in life.

On the other hand, it is much more likely that a male sibling who comes from a family with an autistic child has autism than a female sibling. In this case, a PGD may be used simply to pick the sex of embryos to reduce the probability of birth of the affected sex. Ethically, sex selection is debatable despite the presence of medical indications and would be subject to continuous scrutiny [18].

HLA Typing

Lately, several PGD cases are used for HLA typing, as well as for monogenic testing for a particular condition, for the purpose of aiding the treatment strategies for an older sibling or relative. This is done by selecting embryos with identical HLA types to that of a suffering child from particular hematopoietic illnesses such as leukemia, Fanconi anemia, and thalassemia that require the donation of cells from a "savior sibling." In this method, the donor's umbilical cord cells may be grafted to the affected child. This method has also been effectively employed in a group of immunodeficiency syndromes [19]. This use of PGD is referred to as HLA typing. This method of PGD was first recorded by Verlinsky, and overall 23 per cent of IVF clinics conducted PGD for HLA typing in combination with genetic analysis to ensure that the baby was also free from the genetic disorder affecting the elder brother or sister [28]. It is unknown how many PGD cycles were performed worldwide for HLA matching and how many children were cured as a result of this process. Two European facilities recently released data on 110 children born following HLA matching via PGD. PGD with HLA typing for a subsequent hematopoietic stem cell transplant is only allowed if all other options (such as bone marrow transplantation from an unrelated donor) are either insufficient or not feasible, that is, if no unrelated donor who is compatible with HLA can be located. The National Board of Health must review each case for acceptance after receiving an application from the pediatrician who is treating the sick kid. Since the law was passed in 2004, 12 families in Denmark have been approved for PGD matching [29].

There are ethical implications of using PGD for HLA typing to conceive a child who could serve as a stem cell donor for a sibling in need. These implications are complex, and there are multiple arguments to be made on such a topic. PGD would only provide a grantee for the match and spare the family from considering termination or counting the pregnancy with a child that is not a match for their sibling. There are various ethical concerns, including the potential instrumentalization of the donor child, the best-interest standard, and the psychological impact on the donor child. Critics argue that creating a child solely as a means to save another undermines the child's intrinsic worth. However, as long as the future child's well-being is considered, this may be morally acceptable. Another major point to consider is whether creating a donor child is in the best interest of the sick child; then it can be justified. The dynamic relationships within the family also play a crucial role. Balancing the risks against the benefit is a daily approach in the medical field; it is important to consider the benefit gained from such a decision. In conclusion, conceiving a child to save another can be morally defensible if the future child's rights and well-being are respected, emphasizing the need for further examination of such ethical dilemmas in broader contexts [30].

Limitation of PGD and ethical concerns

PGD can generate false positive and false negative results. There are several causes for misdiagnosis, ranging from human errors, mislabeling of samples, misidentification of embryos or tubes, and transfer of the wrong embryo. Then there are the limitations of specific technologies such as ADO or contamination from cumulus cells or other sources. Misdiagnosis is a problem that some couples may face, though the possibility is extremely low. It may affect parents’ decisions regarding the procedure [16,31].

However, there are some risks of IVF itself, which include premature labor, maternal hemorrhage, C-section, high blood pressure with pregnancy, and diabetes in pregnancy. Psychological, medical, and financial costs are significant too [22]. Many couples faced unexpected emotional and physical distress, including loss of intimacy and control during the insemination process, and feel compelled to keep their PGD experiences private due to cultural stigma. The experience of PGD also affects relationships, with some couples feeling a deep attachment to their embryos regardless of health status [32].

The other major ethical dilemma facing PGD is sex selection, with requests arising from two main groups: parents wanting a male firstborn due to cultural preferences. Parents seeking the opposite gender after having multiple children of the same gender. Gender selection is viewed as a less compelling reason for embryo selection, with potential societal implications if practiced widely, such as skewed sex ratios seen in countries like China and India. The American Society of Reproductive Medicine (ASRM) initially discouraged PGD for gender selection; later, it accepted it for gender variety but not for firstborns. The committee noted a lack of evidence supporting the necessity of gender variety to justify embryo creation and destruction. Acceptable uses of PGD might include families desiring a balance of genders or those who experienced the loss of a child. This issue is especially relevant in Saudi Arabia, where the most frequent use for PGD is sex selection (57.7%) [1].

Another issue that arises with PGD is one of regulations and guidelines. A study conducted in the Gulf Cooperation Council (GCC) countries showed that although there have been some regulations and guidelines for the IVF centers in all the GCC countries, their implementation is left to the discretion of the treating center [33]. The high cost of testing, new infrastructure, and low returns on investments were the reasons for underutilization of the PGD facilities in the GCC countries. With an average cost of an IVF/ICSI cycle in the GCC countries of approximately $3,000-5,000 with an additional $1,500-2,000 for each implantation. The Ministry of Health in Saudi Arabia has provided clear regulations for implementing the law of fertilization, utero-fetal, and infertility treatment units. Regarding the legalization of IVF centers to practice, we notice that the Saudi Arabian government has a higher score than other GCC countries and has a higher score than corresponding centers. This could be due to strong legislative measures imposed by the Saudi government for licensing. Moreover, the standard deviation is large for data obtained from the centers [33].

Another issue is about the future of PGD against the background of recent technology in sequencing and bio-information advances, which would most certainly allow rapid production of even more sequence information from embryos. The purposes and role of the PGD in IVF should be more specified and confirmed; otherwise, the technology can be used to assess non-medical features such as height, memory, color of hair and eye, or athletics. PGD can be regarded without a focused objective as a tool for choosing the "perfect" child and raising the concept of "designer babies" [10]. Even as PGD becomes increasingly common, only a small percentage of people use assisted reproduction since accessing embryos through IVF is invasive and expensive, and for some people, it raises serious ethical problems. As a result, patients only seek PGD when there are sufficient causes, insurance coverage, and service availability [23].

## Conclusions

Finally, the importance of some advanced genetic diagnostic tools is emphasized by increased diseases and by the high percentage of consanguineous marriages in Saudi Arabia. The relation between consanguineous marriages and genetic disease is evident in a wide spectrum of genetic disorders, particularly autosomal recessive disorder. Initially, PGD was restricted to detection of X-linked disorders, though modern techniques such as PCR, FISH, aCGH, and NGS have transformed it into an effective approach towards handling diverse gene-related matters. Improved IVF results and reduced prevalence of genetic disorders are the advantages brought about by PGD; however, there are also disadvantages like high costs incurred by parents, limited access to PGD services for couples, and ethical concerns, among others. Thus, distinct measures must be put in place to address these issues while ensuring that the technique is used ethically and fully benefits affected families. These include comprehensive pre- and post-PGD counseling to help families navigate ethical dilemmas and emotional challenges as well as ensuring that all parents seeking PGD fully understand the procedure, risks, and implications for the resulting child, including emotional and psychological impacts.

It is justifiable to advocate for policies that require insurance companies to cover PGD as part of fertility treatments, especially for families with hereditary diseases. Also, government grants aimed at supporting low-income families seeking PGD, similar to those available for other medical treatments, have to be explored. Research is still needed to establish the economic impact of PGD on healthcare systems, including potential cost savings from preventing hereditary diseases. Research is also needed on the long-term psychological effects on children conceived via PGD, particularly regarding their understanding of their conception and donor status.
